# Editorial: Insight in pharmacological treatment of pain – 2023

**DOI:** 10.3389/fpain.2025.1662611

**Published:** 2025-08-06

**Authors:** Robert Gyula Almasi

**Affiliations:** ^1^ Clinical Center, Pécs University, Pécs, Hungary; ^2^ Pecsi Tudomanyegyetem Klinikai Kozpont, Pécs, Hungary

**Keywords:** elderly pain management, spinal pain, vulvodynia treatment, adjuvant pain medications, opioid misuse, multimodal pain management, pain pharmacological treatment

**Editorial on the Research Topic**
Insight in pharmacological treatment of pain – 2023

Based on the title of the topic, we aimed to gain insight into new treatment options in the field of pain management, which have successfully alleviated pain in individual patients and could be promising options for many others.

We live in a fast-paced world, the population is growing, and life expectancy is increasing. In an ageing world, social systems and economies may be burdened by the care of people with advanced age and comorbidities. Older age can mask many symptoms, but similar symptoms can be the constellations of manifestations of two comorbidities. This clinical overlap poses significant challenges in diagnosis and management, as distinguishing the background cause of the symptoms becomes increasingly complex in elderly patients. The presentation of pain, mood disturbances, and cognitive changes may reflect the simultaneous influence of multiple underlying conditions. Treatments aimed at one symptom—such as an analgesic or selective norepinephrine reuptake inhibitor (sNRI) prescribed for pain—can alleviate symptoms of a separate disorder, highlighting the interconnected nature of neuropsychiatric and somatic processes in advanced age.

In aging patients, when developing cerebral circulatory and degenerative disorders cause damage to brain areas responsible for the functioning of top-down pain modulation systems—such as the frontal cortex or prefrontal area—the pain-alleviating system is disrupted.

Pain symptoms and complaints can develop for no apparent reason, which can be explained by the disintegration of the pain modulatory system. In many cases, painful complaints are associated with depression in the older adults, and these patients visit the doctor with overlapping complaints and symptoms typical of chronic pain. It is very common for these patients to struggle with sleep disorders, compliance problems, self-esteem disorders, family conflicts, misunderstanding, distancing, aggression, anxiety, depression, or suicidal ideations. With the use of antidepressants, these signs and symptoms can improve quickly, which is why many antidepressants are used for pain relief. A co-administration of relatively novel sNRI atomoxetine used for attention deficit hyperactivity disorder (ADHD) and the anti-Parkinson pramipexole could be a new alternative way to treat chronic painful conditions, as Kasahara et al. demonstrated in their case report.

Anticonvulsants are another type of agent effectively used in neuropathic pain, although their side effect profiles are suboptimal. It is especially exciting to read Ergisi et al. narrative synthesis of research on the effect of topical gabapentin in patients with vulvodynia.

Opioids are very effective analgesics; their efficacy has been proven in the treatment of severe or cancer pain. However, due to their poor side effect profile and potential for abuse, their routine use should be reconsidered. While cognitive disorders, frailty, and signs of deterioration predominate in the elderly, younger adults tend to struggle with existential issues, mid-life crises, and relationship problems. Chronic pain patients are prone to illicit opioid use or medication misuse with doctor-seeking behaviour. It can be challenging for practitioners to evaluate individual risk factors for opioid use disorders and avoid misuse of prescription opioids and prevent illicit opioid use. In the article published by Galán et al., we are provided with guidance indicating that Spanish pain specialists agree on the risk factors that can lead to opioid misuse.

Any attempt to eliminate the use of opioids can be helpful in preventing side effects and improving the opioid crisis. Banik et al. questioned the outcomes of human studies and investigated the effects of multimodal drug treatment and dexamethasone on surgical incisions in an animal model. It is heartwarming that this was studied with an opioid-free combination of analgesics and adjuvants. Their study can encourage clinicians to reevaluate their results in this field, which is, after all, one of the aims of translational medicine.

One of the biggest burdens for the individual, the family, and the national economies is back pain. In an attempt to avoid surgery, interventions with anesthetics were regarded as a useful tool to clarify the diagnosis and treat the suspected structural damage. We now know that the effect of surgery and intervention is far below expectations. Spinal surgeries are currently performed only with a rather narrow indication, even in the case of obvious morphological abnormalities. Today, nonpharmacological and nonmedical therapies are the primary focus of pain specialists. Regular exercise, stretching, yoga, lifestyle changes, physical treatments, psychological approaches may help more to solve problems. Since pain is a multidimensional phenomenon, the evaluation and treatment of pain is also multimodal. The interventions used as part of multimodal analgesia provide a lot of help to the patient. It is difficult to estimate how many types of drugs and agents have been evaluated so far in spinal-pain research. In most cases, the underlying morphological problem does not correspond in a one-to-one manner with the severity of pain experienced. Despite this, the number of spinal interventions is not decreasing, indicating the efforts of pain specialists to help their patients. Corticosteroids relieve pain and inflammation; therefore, these agents are included in the palette of pain medicine providers. The effectiveness of corticosteroid injections is difficult to prove with well-designed studies because patients are heterogeneous in terms of demographics, morphology and psychosocial background. Moreover, pain specialists use various types of steroids, at different concentrations and in different volumes, employing a range of methodologies. Considering one of the features of corticosteroids—whether they are particulate or nonparticulate—certain techniques, such as transforaminal injection, pose risks. In regional anesthesia practice, dexamethasone is regarded as an effective adjuvant that extends the duration of action of local anesthetics in patients undergoing peripheral nerve blockade. Antibiotics, analgesics, adjuvants, and contrast materials are also included in the armamentarium of pain clinicians' daily practice. The interventions are often performed in patients with other health conditions that require some form of pharmacotherapy. The possible interactions should not only be considered, but also discussed with patients. The plethora of agents used in the context of spinal intervention is provided in the review by Torralba et al.

In this topic, pharmacotherapy stands as an indispensable ally that empowers clinicians to deliver individualized relief and improve the quality of life of those burdened by pain.

